# Cordopexie par voie combinée endoscopique et externe dans le traitement des paralysies récurrentielles post-thyroïdectomie

**DOI:** 10.11604/pamj.2024.49.52.42060

**Published:** 2024-10-23

**Authors:** Sandra Yowa Muya, Cheikh Ahmedou Lame, Birame Loum, Thierno Boubacar Diallo, Cheikhna Ba Ndiaye, Khady Marie Agnes Diouf, Aly Toure, Charles Latyr Diagne, Mame Rouba Ndiaye, Bamba Sissoko

**Affiliations:** 1 Service d'ORL et de Chirurgie Cervico-Faciale, Hôpital Principal de Dakar, Dakar, Sénégal

**Keywords:** Cordopexie, paralysie récurentielle bilatérale, thyroidectomie, Cordopexy, recurrential bilateral paralysis, thyroidectomy

## Abstract

La paralysie récurrentielle bilatérale en adduction est une complication grave de la chirurgie thyroïdienne, dont la prise en charge est un véritable challenge pour le chirurgien oto-rhino-laryngologiste (ORL). La cordopexie par voie combinée, endoscopique et externe, offre une possibilité de lever la dyspnée laryngée et de rétablir une voix acceptable tout en préservant l'intégrité anatomique des structures laryngées. Il s'agit d'une étude observationnelle descriptive de type transversale avec une collecte de données rétrospective, menée de janvier 2012 à avril 2023, portant sur 7 patients reçus pour paralysie récurrentielle bilatérale post-thyroïdectomie et ayant bénéficié d'une cordopexie par voie combinée. L'évaluation des résultats était faite sur la base de l'échelle de satisfaction de Likert. Tous les patients étaient de sexe féminin avec un âge moyen de 49 ans. La dyspnée était retrouvée chez tous; la nasofibroscopie visualisait des cordes vocales en adduction paramédiane dans 4 cas et médiane dans 3 cas. Une cordopexie par voie combinée avait été réalisée à droite dans 42,9% des cas et à gauche dans 57,1% des cas. L'évaluation du degré de satisfaction des patients par l'échelle de Likert retrouvait, sur le plan respiratoire: 2 patients très satisfaits et 3 patients satisfaits. Un patient était très satisfait et 3 étaient satisfaits de la qualité de leur voix. Deux patients étaient insatisfaits de leur respiration. La cordopexie par voie combinée, endoscopique et externe, est une technique simple, réversible, minimale invasive qui peut être indiquée dans la prise en charge des paralysies récurrentielles bilatérales post-thyroïdectomie avec des résultats satisfaisants.

## Abstract (en)

Bilateral adducted recurrent paralysis is a serious complication of thyroid surgery. The management of such conditions is a real challenge for ENT surgeons. Combined endoscopic and external cordopexy offers a way to alleviate laryngeal dyspnea and restore acceptable voice quality while preserving the anatomical integrity of laryngeal structures. From January 2012 to April 2023, we conducted a cross-sectional descriptive observational study with retrospective data collection, including 7 patients with bilateral post-thyroidectomy recurrent paralysis who had undergone combined cordopexy. Outcomes were assessed using the Likert satisfaction scale. All patients were female, with a mean age of 49 years. Dyspnea was observed in all patients. Nasofibroscopy showed paramedian adduction of the vocal cords in 4 cases and median adduction in 3 cases. Combined cordopexy was performed on the right in 42.9% of cases and on the left in 57.1%. Patient satisfaction, assessed using the Likert scale, showed that, in terms of respiratory outcomes, 2 patients were very satisfied and 3 were satisfied. One patient was very satisfied and 3 were satisfied with the quality of their voice. Two patients were not satisfied with their breathing. Combined endoscopic and external cordopexy is a simple, reversible, minimally invasive technique that can be used, in the management of bilateral post-thyroidectomy recurrent paralysis, with satisfactory results.

## Introduction

La paralysie récurrentielle (PR) est une des complications les plus fréquentes pouvant survenir au décours d'une chirurgie thyroïdienne. Elle peut être bilatérale en adduction, se manifestant essentiellement par une symptomatologie suggestive d'une obstruction des voies aériennes supérieures mettant en jeu le pronostic vital du patient [1] et nécessitant une prise en charge urgente qui se résume la plupart du temps par la réalisation d'une trachéotomie [2]. La chirurgie d'élargissement de la fente glottique demeure le traitement de choix et la cordopexie ou latéro-fixation de la corde vocale est une option. L'objectif de cet article était d'évaluer les résultats d'une technique de cordopexie par voie combinée (CVC), inspirée de celle décrite par Ejnell en 1984 [3] et adaptée à notre milieu d'exercice où le nombre de praticiens ORL est faible avec un plateau technique limité.

## Méthodes

**Conception de l'étude:** il s'agissait d'une étude observationnelle descriptive de type transversale avec une collecte de données rétrospective qui a été réalisée au sein du service d'ORL et de Chirurgie Cervico-Faciale de l'Hôpital Principal de Dakar (ORL-CCF/HPD).

**Cadre de l'étude:** la ville de Dakar est la capitale de la république du Sénégal située à l'extrémité occidentale de l'Afrique, sur la presqu'ile du Cap-Vert ; à 705 Km au nord-ouest de Conakry et à 408 Km au sud-ouest de Nouakchott ; à 14° 43' 55'' nord et 17° 27' 26'' ouest. Sa population est estimée à environ 1 250 000 habitants; elle comprend plusieurs hôpitaux publics dont l'Hôpital Principal de Dakar qui est un hôpital d'Instruction des Armées du Sénégal géré par les Forces Armées Sénégalaises sous la tutelle du Ministre de la Santé et de l'Action Sociale. Cet hôpital est un élément central du groupe hospitalier militaire dakarois néanmoins il demeure un établissement public de santé avec une mission de service public pourvu d'un plateau technique adéquat pour une meilleure prise en charge de patients. Il comprend un service d'ORL et de Chirurgie Cervico-Faciale (ORL-CCF) organisé de manière à prendre en charge toutes sortes de pathologies de cette sphère, faisant de lui un centre préférentiel de référence pour les patients, provenant de la ville de Dakar et des alentours, nécessiteux de soins spécialisés dans le domaine.

**Variables:** cette étude a utilisé des variables explicatives tels que le sexe et l'âge. La paralysie récurrentielle bilatérale en adduction (PRBA) a été classée en type paramédiane ou médiane en fonction de la position des cordes vocales durant la nasofibroscopie préopératoire.

**Figure 1:**
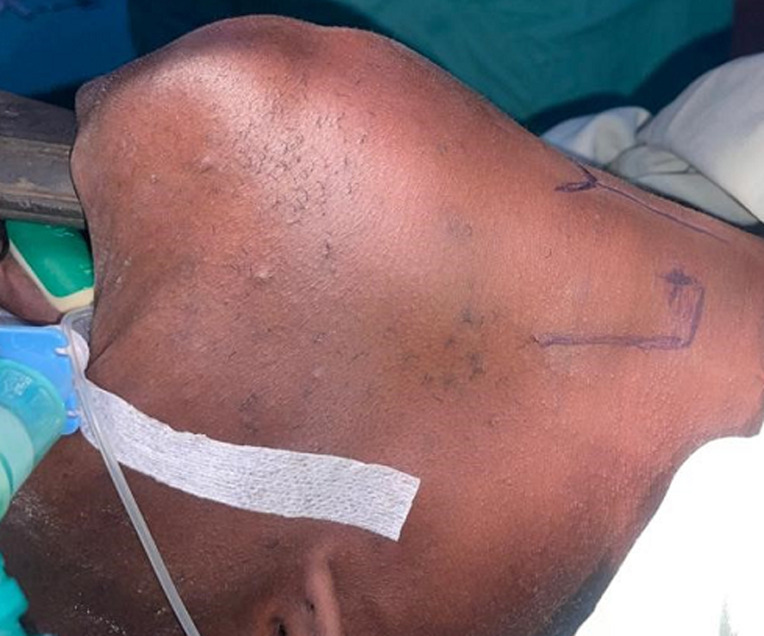
marquage des repères anatomiques superficiels

**Figure 2:**
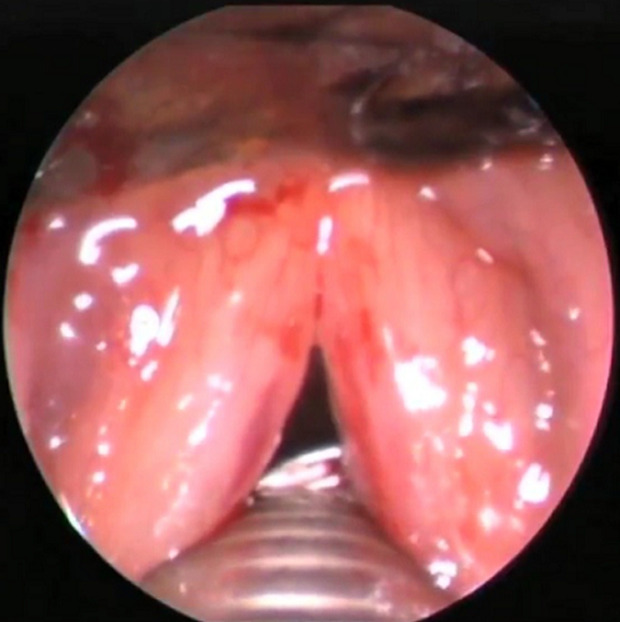
vue endoscopique montrant la première aiguille placée sous le plan glottique

**Figure 3:**
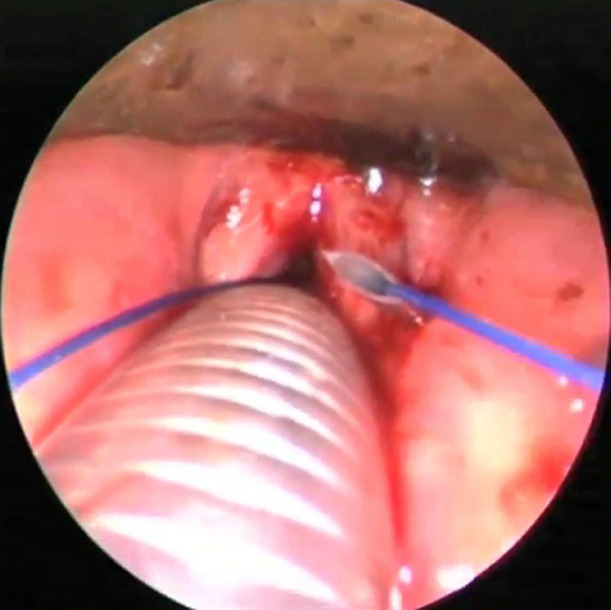
vue endoscopique montrant la deuxième aiguille placée en sus-glottique et passage du fil

**Figure 4:**
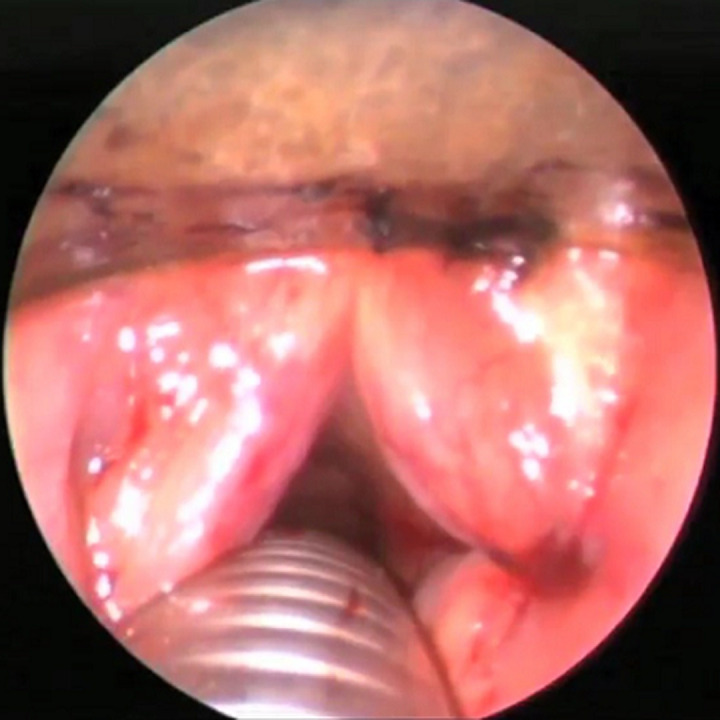
vue endoscopique montrant la boucle de pexie et la fente glottique élargie en fin d'intervention

La technique chirurgicale d'Ejnell modifiée avait été utilisée chez tous les patients et se déroulait comme suit : le chirurgien opère avec une aide et une infirmière circulante se chargera de les habiller et de servir les instruments; un endoscope rigide 0° (diamètre 2,7 ou 3 mm, longueur 30 à 36 cm) est relié à une colonne vidéo (caméra, moniteur et source de lumière froide). Un matériel de laryngoscopie directe en suspension, 2 aiguilles de cathéter veineux ou trocards 16G et un fil prolène 2/0 sont nécessaires; le patient est allongé en décubitus dorsal, un billot placé sous les épaules. Le chirurgien se place du côté de la pexie, l'aide opérateur (endoscopiste) à la tête du patient. Le moniteur vidéo en face de l'endoscopiste; la procédure chirurgicale est réalisée sous anesthésie générale avec intubation orotrachéale, ou faite par l'orifice de trachéotomie. Une sonde de petit calibre est utilisée à défaut de jet ventilation; la CVC se fait selon les étapes suivantes: après marquage des repères anatomiques superficiels (Figure 1), l'appareillage de suspension est mis en place. L'examen endoscopique permet de bien visualiser le larynx. La première aiguille de cathéter 16G est introduite dans le larynx par voie transcutanée, à la jonction entre les deux-tiers supérieurs et le tiers inferieur de la lame du cartilage thyroïde; l'aiguille est ensuite repérée sous le plan glottique (Figure 2); un fil prolène 2/0 est passé dans l'aiguille, puis récupéré en endoluminal; la deuxième aiguille de cathéter est introduite, environ 5 mm au-dessus de la première suivie de son repérage en endoluminal au-dessus de la corde vocale (Figure 3); le fil en endolaryngé est enfilé dans la deuxième aiguille pour être extériorisé sur la peau où le nœud est réalisé puis enfoui en sous-cutané (Figure 3); la boucle ainsi réalisée autour de la corde vocale, idéalement à la jonction des deux-tiers antérieurs et du tiers postérieur, exerce une traction sur celle-ci, autorisant une fente glottique acceptable et compatible avec une respiration correcte (Figure 4). Un contrôle endoscopique était réalisé en fin d'intervention et une nasofibroscopie était faite à 1 mois, 3 mois et 6 mois postopératoire. L'évaluation à long terme avait été faite sur la base de l'appréciation, par le patient, de la qualité de sa voix et de sa respiration grâce à une échelle de satisfaction décrite par Likert avec 4 points répartis comme suit : très satisfait = 4; satisfait = 3; ni satisfait ni insatisfait = 2; insatisfait = 1

**Collecte des données:** cette étude était observationnelle descriptive de type transversale avec une collecte de données rétrospective qui a été réalisée au sein du service d'ORL et de Chirurgie Cervico-Faciale de l'Hôpital Principal de Dakar (ORL-CCF/HPD) de janvier 2012 à avril 2023, en se concentrant sur les individus présentant une paralysie récurrentielle bilatérale en adduction post thyroïdectomie et ayant bénéficié d'un traitement chirurgical sous forme de cordopexie par voie combinée endoscopique et externe.

**Ressources et mesure des données:** les données ont été extraites des dossiers médicaux ainsi que des registres des compte rendus opératoires des patients ayant bénéficié d'une CVC indiquée devant une paralysie récurrentielle bilatérale en adduction. Les variables liées aux caractéristiques sociodémographiques, liée à la durée d'évolution des symptômes avant la prise en charge, aux antécédents de chirurgie thyroïdienne et de trachéotomie, les aspects cliniques et la prise en charge chirurgicale à type de CVC ainsi que le suivi post opératoire et les modalités évolutives, avec l'évaluation par l'échelle de Likert, ont été consignés sur une fiche de récolte de données avant leur saisie dans Microsoft Excel et Word 2013, manuellement et de manière rigoureuse afin d'en garantir la cohérence.

**Participants à l'étude, recrutement et taille de l'échantillon:** ils ont été inclus tous les patients ayant bénéficié d'une CVC indiquée devant une paralysie récurrentielle bilatérale en adduction (PRBA) qui avait été préalablement confirmée par la nasofibroscopie chez des patients qui avaient présenté des symptômes évocateurs à type de dyspnée laryngée associée ou non à une dysphonie survenue dans les suites d'une chirurgie thyroïdienne. Tous les cas répertoriés de PRBA ayant bénéficié d'une CVC durant la période d'étude ont constitué notre échantillon, regroupant ainsi un total de 7 patients.

**Gestion et analyse des données:** nous avons utilisé Microsoft Word et Excel 2013 pour l'analyse des données. Une variable quantitative a été décrite par la moyenne. Les variables qualitatives ont été présentées sous forme de fréquence et de pourcentage.

**Approbation éthique et consentement éclairé à participer:** tous les patients avaient fourni un consentement éclairé avant leur traitement chirurgical. Nous avons obtenu l'approbation de tous les patients afin d'utiliser les données recueillies au cours de leur suivi à des fins de recherche, dans le strict respect de leur anonymat. Nous avons également obtenu une autorisation de recherche du Médecin-chef, Directeur de l'hôpital pour la récolte des données, garantissant le respect des normes de confidentialité des personnes concernées.

## Résultats

Nous avons ainsi colligé 07 cas de paralysie récurrentielle bilatérale post-thyroïdectomie ayant bénéficié d'une cordopexie par voie combinée. Tous les patients étaient de sexe féminin avec une moyenne d'âge de 49 ans avec des extrêmes allant de 24 à 66 ans. La dyspnée était retrouvée chez tous les patients. Les 6 présentaient tous une dysphonie, La dernière patiente était porteuse d'une trachéotomie.

La durée moyenne d'évolution des symptômes était de 73,14 mois avec des extrêmes allant de 5 mois à 324 mois et le délai moyen de prise en charge dans le service était de 3,57 mois avec des extrêmes allant de moins d'1 mois à 15 mois. A la nasofibroscopie, les cordes vocales étaient immobiles en adduction, en position paramédiane dans 4 cas et médiane dans 3 cas. Sur le plan thérapeutique, une cordopexie par voie combinée avait été réalisée à droite dans 42,9% des cas (3 patients) et à gauche dans 57,1% des cas (4 patients).

Les résultats de la procédure étaient appréciés sur la base de l'amélioration des signes fonctionnels. Le degré de satisfaction de nos patients était évalué, avec un recul minimal de 6 mois, sur la base de l'échelle de satisfaction de Likert; elle retrouvait sur le plan respiratoire 2 patients très satisfaits et 3 patients satisfaits. Un patient était très satisfait et 3 patients étaient satisfaits de la qualité de leur voix (Tableau 1). Le taux de réussite de la cordopexie par voie combinée était ainsi estimé à 71,4% (5 cas) avec 2 cas d'échec. Ce taux d'échec de 28,6% était lié à un arrachement précoce du fil de pexie chez un patient et à la présence d'un granulome trachéal chez un autre patient.

**Tableau 1:** évaluation du degré de satisfaction des patients en période postopératoire tardive selon l'échelle de Likert

Degré de satisfaction	Respiration	Voix
Très satisfait	2 (28.6%)	1 (14,3%)
Satisfait	3 (42,8%)	3 (42,8%)
Ni satisfait ni insatisfait	0 (0,00%)	1 (14,3%)
Insatisfait	2 (28,6%)	2 (28,6%)
**Total**	7 (100%)	7 (100%)

## Discussion

La paralysie récurrentielle bilatérale en adduction est une complication grave de la chirurgie thyroïdienne [1,4,5]. Sa prise en charge demeure un challenge dans notre contexte d'exercice. La chirurgie d'élargissement de la fente glottique est une des options thérapeutiques. Plusieurs techniques ont été décrites à ce jour. La chirurgie endoscopique au laser est actuellement en vogue, permettant de réaliser cordotomies, cordectomies ou cordo-arytenoïdectomies [5-8]. La chirurgie au laser peut être associée à la latéralisation de la corde vocale [9], mais elle reste encore très peu accessible dans nos pays. Dans les pays en voie de développement, la chirurgie par voie externe garde toute sa place, permettant de réaliser des arytenoïdopexies, des arytenoïdectomies associées ou non à des cordopexies [10,11]. Elle donne des résultats satisfaisants sur le plan respiratoire, cependant la voix n'est pas toujours de bonne qualité, en plus des cicatrices parfois inesthétiques, liées à la voie d'abord.

Ejnell a décrit, en 1984, une technique chirurgicale de latérofixation de la corde vocale par mise en place d'un fil de suture en boucle autour de la corde vocale, après exposition cervicale de la lame du cartilage thyroïde par dissection [3]. Lichtenberger a développé, en 1983, un porte-aiguille endo-extralaryngé pour guider un fil externe à l'intérieur du larynx et réaliser ainsi l'abduction de la corde vocale en fixant le fil sur un taquet en plastique calé contre le cartilage thyroïde. Il a démontré, en 2002, que la latérofixation des cordes vocales permettait d'éviter la morbidité liée à la trachéotomie dans le traitement des paralysies bilatérales des cordes vocales [12,13].

Plusieurs modifications de ces techniques ont été effectuées au fil du temps, faisant recours à un abord cervical moins délabrant, et donnant des résultats satisfaisants sur le plan respiratoire et vocal [14-18]. La procédure que nous décrivons est une technique simplifiée, utilisant 2 aiguilles de cathéter passées par voie percutanée pour introduire le fil et réaliser la boucle autour de la corde vocale avant la latérofixation ; telle que décrite par certains auteurs, utilisant également des aiguilles [19,20]. Les résultats sont satisfaisants sur le plan respiratoire et sur le plan phonatoire. Cette technique simple, peu invasive, réversible et reproductible peut être une alternative à la trachéotomie. En cas d'échec, elle laisse la place aux autres options thérapeutiques.

## Conclusion

La paralysie récurrentielle bilatérale post-thyroïdectomie pose des difficultés thérapeutiques. La technique utilisée chez nos patients est simple de réalisation, très peu invasive et efficace. Elle est reproductible et réversible. Elle donne des résultats satisfaisants et est particulièrement adaptée à notre contexte d'exercice.

### Etat des connaissances sur le sujet

- La latéralisation de la corde vocale s'adresse aux paralysies récurrentielles bilatérales en adduction, toutes causes confondues;
- C'est une chirurgie satisfaisante du fait qu'elle permet la préservation des structures anatomiques du larynx.

### Contribution de notre étude à la connaissance

- Notre étude décrit les résultats fonctionnels de cette chirurgie par voie combinée endoscopique et externe;
- Elle décrit une prise en charge à l'aide d'instruments de base en chirurgie laryngée ORL et en utilisant les moyens à la disposition de tous;
- Une prise en charge par cette procédure permet d'éviter une chirurgie lourde et délabrante; elle est réversible et offre une possibilité de reprise ou de recours à d'autres techniques en cas d'échec.
